# Mindfulness practice correlates with reduced exam-induced stress and improved exam performance in preclinical medical students with the “acting with awareness”, “non-judging” and “non-reacting” facets of mindfulness particularly associated with improved exam performance

**DOI:** 10.1186/s40359-022-00754-3

**Published:** 2022-02-23

**Authors:** Jasmine Heath Hearn, Claire J. Stocker

**Affiliations:** 1 Department of Psychology, Manchester Metropolitan University, Brooks Building, 53 Bonsall Street, Manchester, M15 6GX UK; 2 Phase 1 Lead, Aston Medical School, Aston University, The Aston Triangle, Birmingham, B4 7ET UK

**Keywords:** Medicine, Medical school, Meditation

## Abstract

**Background:**

Medical students demonstrate higher levels of psychological distress compared with the general population and other student groups, especially at exam times. Mindfulness interventions show promise in stress reduction for this group, and in the reduction of cortisol, an established clinical marker of the body’s stress response. This study investigated the relationship of mindfulness to exam-induced stress, salivary cortisol and exam performance in undergraduate medical students.

**Methods:**

A controlled pre-post analysis design with within-groups comparisons. 67 medical students completed the five facet mindfulness questionnaire (FFMQ) and provided saliva samples, from which cortisol was extracted, during group work (control/baseline) and immediately prior to end of year 2 examinations (experimental). Academic performance data was extracted for comparison with measures.

**Results:**

Exam-induced salivary cortisol concentration showed a significant negative relation with exam performance. Total FFMQ score showed a significant positive relation with exam performance and a significant negative relation with exam-induced salivary cortisol. The specific mindfulness facets of acting with awareness, non-judging and non-reacting also showed a positive correlation with exam performance.

**Conclusions:**

This study suggests that there exists an important relationship between mindfulness and the physiological biomarker of stress, cortisol, and this manifests into improved assessment outcomes potentially through healthier, more adaptive coping and stress management strategies. In particular, this study identifies the acting with awareness, non-judging and non-reacting facets of mindfulness to be significantly associated with exam performance suggesting that these may be important facets for clinical educators to target when helping students with mindfulness practice.

## Background

The development of competent and compassionate doctors requires years of medical education. Unfortunately, some of the demands of medical training, including study burden, busy schedules [1] and the assessment process [2], demonstrate detrimental effects on the mental wellbeing of medical students. Recent studies estimate that the prevalence of depression, stress and anxiety in medical students is up to 30%, 49%, 32% respectively [3, 4], which is higher than that of the general population and age-matched peers [5]. Excess stress in the short term may lead to sleeping disturbances and poorer academic performance [6], with exam-induced stress shown to predict academic performance in medical students [7], which may have further adverse effects on students’ academic and emotional wellbeing. This is particularly relevant to early years medical students who tend to be exam-orientated [8, 9]. Chronic stress may increase students’ risk of cardiovascular disease, immune dysfunction, neuroendocrine dysregulation, and subsequent mental health disorders later in life [10]. Furthermore, psychological distress may be detrimental to empathy and optimism in medical students [11, 12], therefore demonstrating the need for effective stress management tools and support for this group of students, particularly in the early years of medical education.

In response to stressors, students often use maladaptive coping strategies such as escape avoidance, in which wishful thinking and behavioral efforts are made to escape or avoid the problem [13]. These avoidance behaviors have the potential to negatively affect academic performance [14]. In contrast, mindfulness is an approach-focused strategy to managing stress, involving non-judgmental awareness of the present moment, as opposed to avoiding negative experiences. Mindfulness-based interventions have been studied in medical students, with promising results in terms of stress reduction [15], and improvements in wellbeing [16]. Likewise, recent evidence has demonstrated that lower perceived stress was associated with greater mindfulness, and that a brief mindfulness intervention in medical training was effective in maintaining lower perceived stress [17]. Further, a recent systematic review concluded that mindfulness is beneficial for reducing stress in medical students [18], thereby demonstrating its efficacy for medical students.

Stressors act upon the hypothalamus–pituitary–adrenal axis (HPA axis) neuroendocrine pathway to secrete cortisol from the adrenal gland. Under normal physiological conditions, cortisol is released in a diurnal cycle, with highest plasma levels 30 min after waking each morning, reducing to an average level an hour after waking and remaining at that level for several hours before decreasing to a low [19]. However, cortisol concentrations show acute increases in response to stressors [20]. Glucocorticoid dysregulation is strongly associated with a wide range of stress factors, and under prolonged stress, cortisol can remain chronically elevated [21]. This causes damaging levels of inflammation in the central nervous system affecting neurological function [22] including hippocampal learning [23]. Increases in salivary cortisol before exams can inhibit both learning processes [24] and reduce exam performance [25]. This acute response makes cortisol a useful clinical marker to determine physiological responses to stress [20]. Demonstrable reductions in cortisol following four days of mindfulness training have been observed in Thai medical students [26]. In contrast, a study in students from a range of faculties at Cambridge University found no relationship between plasma cortisol levels and mindfulness training [27]. These discrepancies might be explained by the measurements of stress being chronologically dissociated from exam scheduling. The present study, therefore, aimed to establish the potential role of mindfulness in medical student stress induced by exams and the impact of mindfulness on exam performance. This may have implications for the management of stress in medical students through mindfulness training before and during exam times and for student support services to help optimize wellbeing and academic attainment, thus providing a foundation for stress management in further clinical training.

## Methods

### Design

This was a cross-sectional study with within- and between-subject assessments. Participants provided measures at baseline (non-stressful, group work condition), thereby acting as their own control, and prior to summative assessments (stressful condition) at the end of year 2.

### Participants

Eligible participants were undergraduate medical students recruited from three cohorts commencing Year 1 studies on three consecutive years (2014–2016). Inclusion criteria were: aged over 18 years of age (no upper age limit), students enrolled on an undergraduate medical training program, and were in their second year of study. All participants were introduced to mindfulness in Year 1 as part of their training and encouraged to adopt the practice, although this was entirely optional. Participation was voluntary, with written informed consent obtained, with 122 students participating in the study and 67 students completing all parts of the study (8 from the 2014 cohort, 40 from the 2015 cohort, and 19 from the 2016 cohort). Students with metabolic disorders or medication that could affect cortisol levels were excluded from participation in the study. Also excluded were participants who had engaged in exercise, eating and drinking anything other than water after 7.30 am as this could have affected salivary cortisol concentration.

### Procedure

At the end of Year 2, participants who consented were approached during a standard group work session in term time, seven days prior to exam day (non-stressful condition) and provided a saliva sample and a rating of their current stress level. After which a demographic questionnaire and the Five Facet Mindfulness Questionnaire-Short Form were administered. Following this, prior to summative assessments (stressful condition) at the end of Year 2, students provided another rating of their current stress level, and a saliva sample. Examinations were written short answer papers, based on 12 clinical vignettes, with a possible ten marks available per vignette. Scenarios are based upon common presentations a student would be expected to see in practice. Saliva sampling and questionnaires were conducted between 8.30 and 9.00 am at both time points, with sleep times of the night before noted to minimize variation of the cortisol concentration between participants and between collection days. Upon completion of the study, participants were provided with a debrief letter detailing the study and reminding them of their right to withdraw until data were anonymized for analysis.

### Measures

#### Demographic characteristics

Of the 67 participants that provided complete data (i.e., baseline and pre-exam measures of perceived stress and saliva samples), 27 (40.3%) were male and 61 (91%) were single. The sample was diverse in ethnicity; 28.4% of the sample were white, 44.8% were Asian, and 13.4% were black/Caribbean/African. Detailed participant characteristics can be found in Table 1. Independent-samples t-tests were conducted at baseline to compare the influence of demographic characteristics on mindfulness, baseline stress and cortisol, pre-exam stress and cortisol, and exam results. There were no significant differences in gender across these outcomes (*p* > 0.05). There were also no significant differences between those who had any previous experience of mindfulness and those who had none.

**Table 1:** Participant demographics

	*N*	*%*
*Gender*		
Male	27	40.3
Female	40	59.7
*Age*		
18–20	23	34.3
21–24	30	44.8
25–29	9	13.4
30–34	4	6.0
35–39	1	1.5
*Marital status*		
Single	61	91.0
Married	2	6.0
Cohabiting	4	3.0
*Ethnicity*		
White	19	28.4
Mixed/multiple	4	6.0
Asian	30	44.8
Black/Caribbean/African	9	13.4
Other	5	7.5

##### Perceived stress

To minimize the time commitment and ensure that students could utilize the time before the exam in the most constructive way possible for them and any risks of further stress/distress in the immediate time prior to examinations, students were asked to provide a stress rating on a 10-point Likert-like scale, with 0 representing no stress at all, 5 representing moderate stress, and 10 representing stress as bad as it could be. This method was selected over other questionnaire methods which capture stress experienced over a longer period e.g., a month in the case of the perceived stress scale [28]. This was anticipated to encourage participation in the study during an already stressful and time-limited period and maximize the likelihood of the response provided reflecting current stress levels at the point of measurement, rather than the score being influenced by other variables. The 10-point Likert-like scale for measuring perceived stress has been validated against increased electromyography activity [29].

##### Salivary cortisol

Cortisol was identified as the appropriate biomarker of stress in this study using the method described by Crosswell and Lockwood [30]. Cortisol was obtained using the Salivette sampling device (Sarstedt, 51,582 Numbrecht, Germany). Participants were instructed to place a cotton swab underneath their tongue for one minute and then transfer the swab to a centrifugation tube with a filter insert. Immediately following collection, samples were spun down for 15 min at 25,000 RPM in a centrifuge held at 4 °C before being frozen at − 80 °C until analysis. Analysis was conducted by ELISA as per the manufacturer’s instructions (cat # 1-3002, Salimetrics, Carlsbad, California, USA) for salivary cortisol measurement. The ELISAs had an intra-assay coefficient of variability of 4.2% and an inter-assay coefficient of variability of 7.4%.

##### Five facet mindfulness questionnaire-short form (FFMQ-SF; Bohlmeijer et al. [31])

The FFMQ-SF consists of 24 items scored on five-point Likert scales ranging from 1 (never/rarely true) to 5 (very often/always true). It measures five factors representing elements of mindfulness: observing, describing, acting with awareness, non-judging of inner experience, and non-reactivity to inner experience, thus analyses can demonstrate which skills are important predictors of symptom reduction. Facet scores range from 5 to 25, apart from the facet of observing, which ranges from 4 to 20. The total maximum score on the FFMQ-SF is therefore 120, with higher scores indicating greater levels of mindfulness. The FFMQ-SF has strong psychometric characteristics, including good reliability with alpha coefficients above α = 0.70 for all facets. The FFMQ-SF demonstrated a reliability of α = 0.86 for the total score, and 0.74, 0.74, 0.77, 0.82 and 0.75 for the five facets of non-reacting, observing, action awareness, describing and non-judging respectfully, indicating good reliability [31]. The short version was utilized to encourage participation by reducing participant burden.

### Statistical methods

Data from two published meta-analyses of research involving the assessment of outcome measures in association with mindfulness [32, 33] concluded that studies involving mindfulness produce mean effect sizes of 0.59 and 0.54, respectively (considered medium sizes, according to Cohen’s (1988) criteria [34]. Based on this, an a priori power calculation was undertaken to establish the sample size required; based on a medium effect size = 0.50, alpha = 0.05 and confidence level = 0.95, the sample size required was n = 67, protecting against Type I error. Data were initially examined for distribution normality and outliers. Baseline and pre-exam salivary cortisol concentration and Perceived Stress scores are presented as means ± SEM standard error and statistically compared by Student’s t-test. Relations of exam-induced stress on exam performance were analyzed by Simple Linear Regression and the effect of mindfulness practice on exam score and stress by Multiple Linear Regression based on Principal Component Analysis, with the predictors and criteria explained in the figure captions. Data were analyzed using Prism 9.1.2.226.

### Ethical considerations

This study was approved by the University School of Science and Medicine Ethics Committee. All students gave their prior written consent for participation in the study and publication of results. So that the data collected were valid and therefore reflective of real-world stressors in a medical students’ life, written examinations were utilised as stressors for this study. However, efforts were made to minimise the time commitment required of participants before examinations, and to ensure that participation in the study did not induce further stress, nor adversely affect examination performance. In line with this, participants were briefed prior to taking part in the study, and the time taken to complete assessments before examinations was reduced to a maximum of ten minutes.

## Results

### Perceived stress and salivary cortisol concentrations are increased in the pre-exam situation

**Fig. 1:**
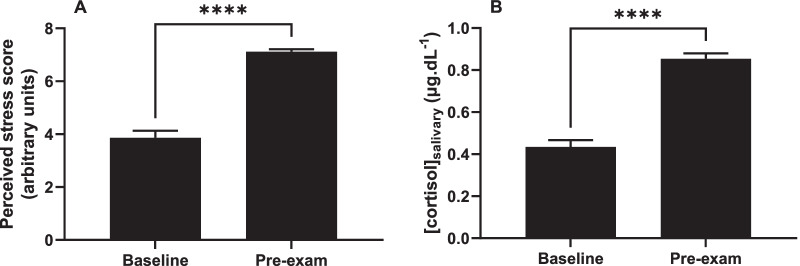
The effect of exam conditions on stress. Baseline and pre-exam levels of perceived stress (**A**) and salivary cortisol concentration (**B**). N = 67. ****p < 0.0001

A paired-samples t-test was conducted to compare perceived stress in the non-stressful condition of group work and the stressful examination condition. There was a significant difference in the scores, indicating that examination conditions are perceived as significantly more stressful than group work conditions (*p* < 0.0001, Fig. 1A). This was confirmed by salivary cortisol levels, which were significantly increased in the examination condition compared with group work (*p* < 0.0001, Fig. 1B).

### Exam performance negatively relates to increase in salivary cortisol levels between baseline and pre-exam time points

**Fig. 2:**
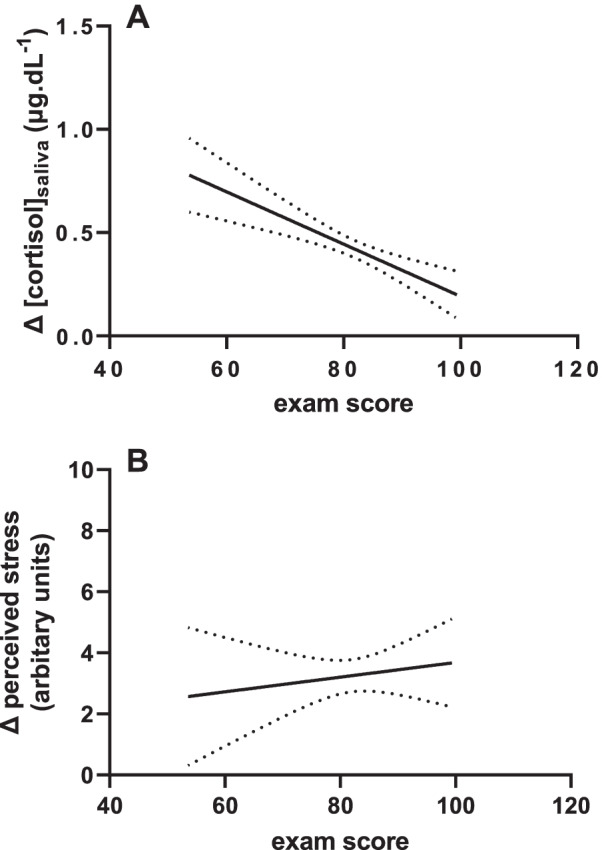
The effect of exam-induced stress on exam performance. **A** Simple linear regression with predictor exam score and criterion exam-induced salivary cortisol significantly deviates from zero (B =  − 0.01265, *p* < 0.0001). **B**. Simple linear regression with predictor exam score and criterion exam-induced perceived stress does not significantly deviate from zero (B = 0.02404, *p* = 0.5369)

No significant correlations were found between exam results, perceived stress scores or salivary cortisol concentration at either baseline or pre-exam, although (Fig. 2). The change in each individual’s salivary cortisol concentration had a significant negative relation with exam score (*p* < 0.0001, Fig. 2A) indicating that the greater the change in cortisol levels between group work and pre-exam conditions, the poorer the exam performance. However, the pre-exam increase in perceived stress did not relate to exam performance (Fig. 2B).

### FFMQ total mindfulness relates positively with exam performance and negatively with the change in salivary cortisol concentration and perceived stress in exam-induced stressful conditions

Regression analysis of exam scores showed significant positive relation with the students’ FFMQ total mindfulness score (*p* < 0.0001, Table 2). Exam scores also showed a significant positive correlation with the students’ FFMQ acting with awareness, non-judging, and non-reactivity scores. The FFMQ total mindfulness score also had a significant negative relation with the change in exam-induced salivary cortisol concentration (*p* = 0.02). However, there was no significant relation between the FFMQ mindfulness scores and students’ exam-induced perceived stress scores (*p* = 0.16).

**Table 2:** The effect of mindfulness practice on exam score and stress

	Criteria					
	FFMQ total mindfulness score	FFMQ observing score	FFMQ describing score	FFMQ acting with awareness score	FFMQ non-judging score	FFMQ non-reactivity score
*Predictors*						
Exam score	B = 0.79 *p* < 0.0001	B = 0.10 *p* = 0.08	B = − 0.07 *p* = 0.17	B = 0.19 *p* = 0.002	B = 0.13 *p* = 0.02	B = 0.24 *p* < 0.0001
Exam-induced perceived stress	B = -0.76 *p* = 0.16	B = − 0.09 *p* = 0.63	B = 0.37 *p* = 0.02	B = − 0.08 *p* = 0.69	B = 0.14 *p* = 0.46	B = − 0.25 *p* = 0.24
Exam-induced salivary cortisol	B = − 13.93 *p* = 0.02	B = − 2.68 *p* = 0.19	B = 1.27 *p* = 0.50	B = − 2.95 *p* = 0.19	B = − 2.11 *p* = 0.33	B = − 4.15 *p* = 0.08

Multiple linear regression analysis standardized regression coefficients (B) and *p* values of FFMQ mindfulness scores with exam score and exam-induced salivary cortisol concentration and perceived stress

## Discussion

In our opportunistic study, healthy medical students were exposed to an objectively stressful situation of an exam, and relationships between exam performance, mindfulness, perceived stress and salivary cortisol in response to this were examined. This study therefore explored whether participants’ changes in subjective experience were associated with stress-related salivary concentrations of cortisol and exam performance, and whether mindfulness experience could buffer these effects. The present study demonstrated that exam conditions elevated cortisol, and increased cortisol was correlated with a reduced exam performance. Importantly, students who scored more highly in the FFMQ questionnaire total score displayed reduced levels of exam-induced salivary cortisol and perceived stress levels immediately prior to exams and performed better in the exam itself than those with lower total FFMQ scores. This suggests that there exists an important relationship between mindfulness and biomarkers of stress, which may manifest into improved assessment outcomes potentially through healthier, more adaptive coping and stress management strategies.

These findings align with a study that found that greater perceived stress before an exam was associated with greater levels of B-cells (another important clinical marker of immune activity that may increase chronic tissue inflammation) [27]. However, in contrast to the present study, that study found no further associations between biomarkers of distress (including cortisol, among others) and mindfulness. Such conflicting evidence demands the need for larger, randomized trials to clearly establish the relationships between variables examined in the present study, and other work in this field.

A novel strength of the present study is the ability to explore the nuance within the measure of mindfulness and individual facets, with results demonstrating that exam scores showed a significant positive correlation with the students’ FFMQ acting with awareness and non-judging scores. Acting with awareness is the ability to act out of quick judgment appropriately to the situation before responding. Acting with awareness may mitigate stress through the prioritization of focus on the present, which may reduce engagement in hypervigilance and attention paid to negative appraisals [35]. A non-judgmental stance towards stress-inducing circumstances such as exams may make students less likely to engage with negative appraisals and catastrophic thinking surrounding the experience, and as such become less prone to increased perceived stress and cortisol excretion. These findings echo previous studies in which both factors were found to mitigate anxiety in medical students [36] and students in general [37], as well as being associated with lower depression severity in general populations and represents potentially important areas for future work to focus on improving. This suggests that these facets of mindfulness, acting with awareness (attending to what is happening in the present) and non-judging (taking a non-evaluative stance toward internal thoughts and feelings), are particularly important skills in managing exam-related stress.

The results of the present study support the suggestion that the integration of mindfulness training as a supportive mechanism in medical school (as suggested by the UK General Medical Council) is warranted and of value. Indeed, multiple medical schools already provide mindfulness resources to support their students [38]. Others have furthered this by integrating mindfulness into their curriculum, such as Monash University in Australia, with studies showing decreased depression and hostility, even pre-exam time, as a result [16]. However, being afforded the choice to participate in mindfulness training may influence its effects. Students were less satisfied with mandatory mindfulness training, compared to voluntary sessions, which suggests that integration into the curriculum may not always be the most effective approach [39]. Further research is required to determine the more efficacious ways to support mindfulness practice in medical students to maximise potential benefits and minimise dissatisfaction or adverse effects. Institutional support is crucial to the successful implementation of mindfulness, and this can take many forms at multiple levels [40]. It is recommended that individual schools consult their student and staff body and elect to offer mindfulness training in ways that best suit their own curriculum and student need and demand. Regardless of the approach medical schools may opt to take, the present study provides further evidence of the potential that mindfulness could offer in improving stress management and academic success.

### Limitations and future research

Cortisol is a reliable biomarker of stress [20], however it is not only secreted in response to stressors, but in a regular diurnal fashion which could be a confounding limitation to the study. The peak cortisol level occurs 30 min after waking and is back to the daily average after 1 h and stays in the average range for several hours [19]. Accordingly, the morning exam time and sampling likely occurred at a time when the diurnal variation is at its most stable and least variable, as has been demonstrated on other studies [19]. Stress-induced salivary cortisol levels are also affected by the menstrual cycle with higher levels during the luteal phase than during the follicular phase [41], possibly influencing the results of this study which did not control for this effect. Further replication work could add value to our understanding of the role of stress, cortisol, and mindfulness in exam performance by exploring variations in cortisol throughout the day of an assessment to establish the extent to which this variable, and the extent of change in cortisol, are associated with mindfulness and exam performance. Variations of stress and responsiveness to coping strategies have been found among different populations [42] and this study may have not been able to account for this possibility due to the relatively small sample size. Covering these factors would add depth to our understanding of the role of mindfulness as a supportive mechanism in managing stress in medical students. Additionally, whilst our controlled pre-post analysis is a strength of the present study, further work could explore the directionality of these relationships in a longitudinal manner. Longitudinal work would be beneficial in establishing the extent to which further training in mindfulness can improve perceived stress, reduce cortisol production in response to stressful events, and improve performance in academic assessments such as medical school exams, and beyond in medical practice. Future studies may also benefit from a multidimensional measurement of perceived stress, rather than the unidimensional 10-point Likert scale employed in this study. As the present study is essentially a pilot conducted immediately prior to a major exam, it was deemed unethical to ask students to participate in a potentially distracting questionnaire. However, whilst this method has been validated in one study [29], it cannot yet be considered robust. This is particularly important considering that acting with awareness can predict lower levels of perceived stress [43], something the present study suggests may also be the case in early year medical students but does not prove. Future studies employing such tools may provide deeper understanding of how respondents are stressed.

## Conclusions

There is an important relationship between mindfulness and exam-induced stress, which may manifest into improved assessment outcomes potentially through healthier, more adaptive coping and stress management strategies. However, whilst physiological stress and exam performance have been demonstrated to be improved by mindfulness practice, the stress that students’ perceive may not be as reliably affected. Therefore, students may benefit from guided mindfulness as part of the curriculum to help them recognize the benefits. Further work would be required to explore this.

## Data Availability

The data are available to all interested researchers upon request. Please contact the corresponding author.

## Abbreviations

FFMQ: Five facet mindfulness questionnaire
FFMQ-SF: Five facet mindfulness questionnaire-short form

## References

[1] Kulsoom B, Afsar NA (2015). Stress, anxiety, and depression among medical students in a multiethnic setting. Neuropsychiatr Dis Treat.

[2] Lyndon MP, Strom JM, Alyami HM, Yu TC, Wilson NC, Singh PP, Lemanu DP, Yielder J, Hill AG (2014). The relationship between academic assessment and psychological distress among medical students: a systematic review. Perspect Med Educ.

[3] Rotenstein LS, Huckman RS, Wagle NW (2017). Making patients and doctors happier—the potential of patient-reported outcomes. N Engl J Med..

[4] Pacheco JP, Giacomin HT, Tam WW, Ribeiro TB, Arab C, Bezerra IM, Pinasco GC (2017). Mental health problems among medical students in Brazil: a systematic review and meta-analysis. Braz J Psychiatry.

[5] Dyrbye LN, Thomas MR, Shanafelt TD (2006). Systematic review of depression, anxiety, and other indicators of psychological distress among U.S. and Canadian medical students. Acad Med.

[6] Sohail N (2013). Stress and academic performance among medical students. J Coll Phys Surg Pak.

[7] Kötter T, Wagner J, Brüheim L, Voltmer E (2017). Perceived medical school stress of undergraduate medical students predicts academic performance: an observational study. BMC Med Educ.

[8] Goldie J, Dowie A, Cotton P, Morrison J (2007). Teaching professionalism in the early years of a medical curriculum: a qualitative study. Med Educ.

[9] Wargent E, Stocker C (2021). Successful instillation of professionalism in our future doctors. MedEdPublish..

[10] McEwan K, Gilbert P, Duarte J (2012). An exploration of competitiveness and caring in relation to psychopathology. Br J Clin Psychol.

[11] Neumann M, Edelhäuser F, Tauschel D, Fischer MR, Wirtz M, Woopen C, Haramati A, Scheffer C (2011). Empathy decline and its reasons: a systematic review of studies with medical students and residents. Acad Med.

[12] Hojat M, Vergare M, Isenberg G, Cohen M, Spandorfer J (2015). Underlying construct of empathy, optimism, and burnout in medical students. Int J Med Educ.

[13] Sandover S, Jonas-Dwyer D, Marr T (2015). Graduate entry and undergraduate medical students' study approaches, stress levels and ways of coping: a five year longitudinal study. BMC Med Educ.

[14] Stewart SM, Lam TH, Betson CL, Wong CM, Wong AM (1999). A prospective analysis of stress and academic performance in the first two years of medical school. Med Educ..

[15] Phang CK, Mukhtar F, Ibrahim N, Keng SL, Mohd SS (2015). Effects of a brief mindfulness-based intervention program for stress management among medical students: the Mindful-Gym randomized controlled study. Adv Health Sci Educ Theory Pract.

[16] Hassed C, de Lisle S, Sullivan G, Pier C (2009). Enhancing the health of medical students: outcomes of an integrated mindfulness and lifestyle program. Adv Health Sci Educ Theory Pract.

[17] Lampe LC, Müller-Hilke B (2021). Mindfulness-based intervention helps preclinical medical students to contain stress, maintain mindfulness and improve academic success. BMC Med Educ.

[18] Daya Z, Hearn JH (2018). Mindfulness interventions in medical education: a systematic review of their impact on medical student stress, depression, fatigue and burnout. Med Teach.

[19] Elder GJ, Wetherell MA, Barclay NL, Ellis JG (2014). The cortisol awakening response—applications and implications for sleep medicine. Sleep Med Rev.

[20] Bozovic D, Racic M, Ivkovic N (2013). Salivary cortisol levels as a biological marker of stress reaction. Med Arch.

[21] McEwen BS (2007). Physiology and neurobiology of stress and adaptation: central role of the brain. Physiol Rev.

[22] Dinkel K, MacPherson A, Sapolsky RM (2003). Novel glucocorticoid effects on acute inflammation in the CNS. J Neurochem.

[23] McEwen BS, Chattarji S (2004). Molecular mechanisms of neuroplasticity and pharmacological implications: the example of tianeptine. Eur Neuropsychopharmacol.

[24] Joëls M (2006). Corticosteroid effects in the brain: U-shape it. Trends Pharmacol Sci.

[25] Lupien SJ, de Leon M, de Santi S, Convit A, Tarshish C, Nair NPV, Thakur M, McEwen BS, Hauger RL, Meaney MJ (1998). Cortisol levels during human aging predict hippocampal atrophy and memory deficits. Nat Neurosci.

[26] Turakitwanakan W, Mekseepralard C, Busarakumtragul P (2013). Effects of mindfulness meditation on serum cortisol of medical students. J Med Assoc Thai.

[27] Turner L, Galante J, Vainre M, Stochl J, Dufour G, Jones PBD (2020). Immune dysregulation among students exposed to exam stress and its mitigation by mindfulness training: findings from a;n exploratory randomised trial. Clinical Trial Sci Rep..

[28] Cohen S, Williamson G, Spacapan S, Oskamp S (1988). Perceived stress in a probability sample of the United States. The social psychology of health.

[29] Luijcks R, Hermens HJ, Bodar L, Vossen CJ, van Os J, Lousberg R (2014). Experimentally induced stress validated by EMG activity. PLoS ONE.

[30] Crosswell AD, Lockwood KG (2020). Best practices for stress measurement: how to measure psychological stress in health research. Health Psychol Open.

[31] Bohlmeijer E, ten Klooster PM, Fledderus M, Veehof M, Baer R (2011). Psychometric properties of the five facet mindfulness questionnaire in depressed adults and development of a short form. Assessment.

[32] Baer RA (2003). Mindfulness training as a clinical intervention: a conceptual and empirical review. Clin Psychol Sci Pract.

[33] Grossman P, Niemann L, Schmidt S, Walach H (2004). Mindfulness-based stress reduction and health benefits. A meta-analysis. J Psychosom Res.

[34] Cohen J (1988). Statistical power analysis for the behavioral sciences.

[35] Vanden Bogaerde A, Derom E, De Raedt R (2011). Increased interoceptive awareness in fear of flying: sensitivity to suffocation signals. Behav Res Ther.

[36] Fino E, Martoni M, Russo PM (2021). Specific mindfulness traits protect against negative effects of trait anxiety on medical student wellbeing during high-pressure periods. Adv Health Sci Educ.

[37] Bodenlos JS, Wells SY, Noonan M, Mayrsohn A (2015). Facets of dispositional mindfulness and health among college students. J Altern Complement Med.

[38] Barnes N, Hattan P, Black DS, Schuman-Olivier Z (2017). An examination of mindfulness-based programs in US medical schools. Mindfulness.

[39] Aherne D, Farrant K, Hickey L, Hickey E, McGrath L, McGrath D (2016). Mindfulness based stress reduction for medical students: optimising student satisfaction and engagement. BMC Med Educ.

[40] Rac T, Chakravarti A (2020). Eight ways to get a grip on implementing mindfulness sessions in medical schools. Can Med Educ J.

[41] Montero-López E, Santos-Ruiz A, García-Ríos MC, Rodríguez-Blázquez M, Rogers HL, Peralta-Ramírez MI (2018). The relationship between the menstrual cycle and cortisol secretion: daily and stress-invoked cortisol patterns. Int J Psychophysiol..

[42] Pawlaczyk M, Siembida J, Balaj K, Rajewska-Rager A (2020). The assessment of stress level, anxiety, depressive symptoms, and defense mechanisms among Polish and English medical students. Ann Gen Psychiatry.

[43] Fong TCT, Wan AHY, Wong VPY, Ho RTH (2021). Psychometric properties of the Chinese version of Five Facet Mindfulness Questionnaire-short form in cancer patients: a Bayesian structural equation modeling approach. Health Qual Life Outcomes.

